# Predicting the Health-related Quality of Life in Patients Following Traumatic Brain Injury

**DOI:** 10.1055/s-0041-1726426

**Published:** 2021-06-17

**Authors:** Thara Tunthanathip, Thakul Oearsakul, Pimwara Tanvejsilp, Sakchai Sae-heng, Anukoon Kaewborisutsakul, Suphavadee Madteng, Srirat Inkate

**Affiliations:** 1 Division of Neurosurgery, Department of Surgery, Faculty of Medicine, Prince of Songkla University, HatYai, Songkhla, Thailand; 2 Department of Pharmacy Administration, Faculty of Pharmaceutical Sciences, Prince of Songkla University, HatYai, Songkhla, Thailand

**Keywords:** traumatic brain injury, heath-related quality of life, EQ-5D-5L

## Abstract

**Background** Traumatic brain injury (TBI) commonly causes death and disability that can result in productivity loss and economic burden. The health-related quality of life (HRQoL) has been measured in patients suffering from TBI, both in clinical and socioeconomic perspectives. The study aimed to assess the HRQoL in patients following TBI using the European quality of life measure-5 domain-5 level (EQ-5D-5L) questionnaire and develop models for predicting the EQ-5D-5L index score in patients with TBI.

**Method** A cross-sectional study was performed with 193 TBI patients who had completed the EQ-5D-5L questionnaire. The clinical characteristics, Glasgow coma scale (GCS) score, treatment, and Glasgow outcome scale (GOS) were collected. The total data was divided into training data (80%) and testing data (20%); hence, the factors affecting the EQ-5D-5L index scores were used to develop the predictive model with linear and nonlinear regression. The performances of the predictive models were estimated with the adjusted coefficient of determination (R ^2^ ) and the root mean square error (RMSE).

**Results** A good recovery was found at 96.4%, while 2.1% displayed an unfavorable outcome. Moreover, the mean EQ-5D-5L index scores were 0.91558 (standard deviation [SD] 1.09639). GCS score, pupillary light reflex, surgery, and GOS score significantly correlated with the HRQoL scores. The multiple linear regression model had a high adjusted R ^2^ of 0.6971 and a low RMSE of 0.06701, while the polynomial regression developed a nonlinear model that had the highest adjusted R ^2^ of 0.6843 and the lowest RMSE of 0.06748.

**Conclusions** A strong positive correlation between the physician-based outcome as GOS and HRQoL was observed. Furthermore, both the linear and nonlinear regression models were acceptable approaches to predict the HRQoL of patients after TBI. There would be limitations for estimating the HRQoL in unconscious or intubated patients. The HRQoL obtained from the predictive models would be an alternative method to resolve this problem.

The Center for Disease Control and Prevention (CDC) has proposed that traumatic brain injury (TBI) is a major public health problem, which can cause morbidity and mortality for adolescents and middle-aged persons in Thailand. 1 2 3 To be specific, road traffic accidents (RTA) have been frequently the main means of TBI. Mortality from RTA has been reported to be 22,487 to 26,312 persons per year. 4 Major disability following TBI in patients ranged from 0.3 to 1.7%, while the mortality rate was reported as 3.2 to 5.2% of all TBIs. 5 6 7 Fulkerson et al studied severe TBI with a Glasgow coma scale (GCS) score of 3 and 4 and found that 56.7% died within the first year following TBI. 8 Furthermore, the CDC reported that severe TBI had lifetime economic costs, which were direct and indirect medical costs and were estimated to be approximately US $76.5 billion in 2010. 9 Hence, the evaluation of the economic burden of TBI should be conducted in all aspects. 10 11 12

Consequently, both qualitative and quantitative human resource losses from posttraumatic long-term sequelae and death have been recognized. The health-related quality of life (HRQoL) has been recognized as the World Health Organization's (WHO) basic principle. HRQoL refers to the individual's perception of well-being in the physical, mental, and social domains. 13

The HRQoL is considered as one of the health outcome indicators, and various HRQoL measurement tools have been used to estimate TBI, such as the quality of life after brain injury overall scale (QOLIBRI-OS) 14 15 and the traumatic brain injury caregiver quality of life (TBI-CareQOL). 16 von Steinbuechel et al measured the HRQoL among 795 patients with TBI using QOLIBRI-OS and found that the extended Glasgow outcome scale (GOS-E) was associated with HRQoL, 14 while Born et al used this questionnaire and reported that severe head injury and injured extremities were associated with a poorer HRQoL. 15 Moreover, Steadman-Pare et al explored the factors associated with HRQoL by using the self-rated quality of life scale. They found that gender, participation in work and leisure, and emotional support were significantly associated with the HRQoL. 17

Currently, no consensus has been reached to measure HRQoL in TBI. The European quality of life measure-5 domain-5 level (EQ-5D-5L) questionnaire is one of the most widely used generic and preference-based instruments to estimate HRQoL in many countries that provide health norms. 18 19 20 Moreover, this instrument has been used for HRQoL measurement in patients with various diseases such as cancer, degenerative diseases, or metabolic diseases. 21 22 23 24

Prior research has studied the health status using the EQ-5D-5L questionnaire in TBI. In detail, Voormolen et al studied the HRQoL measured by the EQ-5D-5L questionnaire and the Rivermead postconcussion symptoms questionnaire which compared between patients with and without postconcussion syndrome (PCS) following TBI. The results demonstrated that mean EQ-5D-5L index scores in the PCS group were significantly lower than the non-PCS group. 25 Ward et al conducted a systematic review and mapping study to build a predictive model from age, gender, comorbidity, extracranial injury, and the Glasgow outcome scale (GOS) for predicting the HRQoL as the EQ-5D-5L index score. 26 However, there is a lack of evidence of the HRQoL assessment using the EQ-5D-5L being directly applied to TBI patients from the literature review. Because of this gap, the present study aimed to assess the HRQoL in patients following TBI using the EQ-5D-5L and develop a model for predicting the EQ-5D-5L index score in patients with TBI.

## Methods

### Study Designs and Study Population

A cross-sectional study was performed to assess the HRQoL using the EQ-5D-5L after the research ethics committee approval (REC.61–116–10–1). The study population included TBI patients who were 18 years or older between October 2018 to March 2020 and admitted to a trauma center in southern Thailand. However, patients were excluded for the following reasons: (1) patients died within 48 hours; (2) patients were not able to perform the EQ-5D-5L by themselves and had no caregiver to complete the questionnaire; (3) foreign patients who were non-Thai or non-English speaking.

Eligible subjects had the purpose and the study protocol explained to them, and all patients who agreed with the study protocol signed the informed consent. Hence, the EQ-5D-5L questionnaire in English or Thai language 26 27 28 was completed by self-reporting or proxy reporting before the patients were discharged from hospital. Additionally, electronic medical records were collected such as the clinical characteristics, treatment, and functional outcomes. After resuscitation, the GCS scores were categorized into mild TBI (GCS score 13–15), moderate TBI (GCS score 9–12), and severe TBI (GCS score 3–8). 4 The functional outcome of the present study was assessed using the GOS when the patients were discharged from hospital. The author used the following GOS categories: 1 = death, 2 = a vegetative state, 3 = severe disability, 4 = moderate disability, and 5 = a good recovery. 29 30 31

### Health-related Quality of Life (HRQoL)

The EQ-5D-5L is an HRQoL questionnaire that was introduced by the EuroQol Research Foundation (EuroQoL). The author was allowed to use the questionnaire by the EuroQoL, which comprised five dimensions which are as follows: mobility, self-care, usual activities, pain/discomfort, and anxiety/depression. Each dimension consisted of five levels of response (no problems, slight problems, moderate problems, severe problems, and extreme problems). 27 The EQ-5D-5L was completed by the patients or caregivers; consequently, their responses were converted to scores based on the method of Pattanaphesaj et al. 28 32 Also, the direct assessment of the HRQoL was performed simultaneously by a visual analogue scale (VAS); however, the EQ-5D-5L index scores were mainly analyzed in the present study.

## Statistical Analysis

The clinical characteristics were calculated from the descriptive data. The categorical variables were described in percentages, while the mean with standard deviation (SD) or median with the interquartile range (IQR) was used for describing the continuous variables.

The continuous variables were compared with the Mann–Whitney U test because of the non-Gaussian distribution, while the GCS and GOS were also compared between the subgroups by one-way analysis of variance (ANOVA) test. A scatter plot was performed for visualizing the relationship between the EQ-5D-5L and independent variables. Spearman's correlation was performed, and multicollinearity was explored when the correlation coefficient was more than 0.8. The variance inflation factor (VIF) and tolerance were used to estimate the multicollinearity. A VIF of more than 10 and a tolerance of less than 0.2 were defined as multicollinearity. 33 34

Eighty percent of the total data was used to develop the predictive model, while the remaining 20% was used to test the model's prediction performance. Therefore, linear regression was performed to identify the variables associated with the EQ-5D-5L index score using linear regression. A threshold *p* value of less than 0.1 was used to estimate the candidate variables for selection in the final model. Multiple regression analyses were also performed using the backward elimination procedure, and statistical significance was observed with a *p* value of less than 0.05. Therefore, a model of the nonlinear regression was achieved compared with the model of linear regression. Moreover, the adjusted coefficient of determination (R ^2^ ) referred to the predictive model's level of performance by explaining the EQ-5D-5L, while the value of the root mean square error (RMSE) was the performance of the model's prediction. The statistical analysis was performed using the R version 3.6.2 software (R Foundation, Vienna, Austria).

## Results

Of the 729 TBI patients enrolled in the study, 193 patients consented and completed the questionnaire. Additionally, four patients were excluded because they were foreign patients who could not communicate in Thai or English languages. The mean age was 39.2 (SD 16.5), with a range of 18 to 78 years, while the median age was 36 (IQR 29). More than two-thirds of the group were male, with road traffic accidents (RTA) being the major cause of TBI (67.9%). After resuscitation, 92.7% of the cases had mild TBI, while 7.2% had moderate-to-severe TBI. Almost all of the cohort had normal pupillary light reflex in both eyes. Mortality was not found in the present study, while 96.4% of all participants had a good recovery. Furthermore, the mean EQ-5D-5L index scores were 0.91558 (SD 1.09639), while the mean VAS score was 94.08 (SD 9.71). The baseline characteristics are presented in Table 1 .

**Table 1:** Demographic data of pediatric TBI ( *n* = 193)

Factor	*n* (%)
**Age group-year**	
< 40	102 (52.8)
≥ 40	91 (47.2)
**Mean age– months (SD)**	39.2 (16.5)
**Median age– months (IQR)**	36 (29)
**Gender**	
Male	120 (62.2)
Female	73 (37.8)
**Mechanism of injury**	
Road traffic injury	131 (67.9)
Nonroad traffic injury	62 (32.1)
**Loss of consciousness**	
No	152 (78.8)
Yes	41 (21.2)
**Amnesia**	
No	164 (85.0)
Yes	29 (15.0)
**Vomiting**	
No	153 (79.3)
Yes	40 (20.7)
**Scalp hematoma/laceration**	
No	83 (43.0)
Yes	110 (57.0)
**Bleeding per ear/nose**	
No	186 (96.4)
Yes	7 (3.6)
**Hypotension episode**	
No	190 (98.4)
Yes	3 (1.6)
**Seizure**	
No	190 (98.4)
Yes	3 (1.6)
**GCS score**	
13–15	179 (92.7)
9–12	7 (3.6)
3–8	7 (3.6)
**Mean GCS**	14.46 (1.69)
**Pupillary light reflex**	
Nonreact pupils	6 (3.1)
React pupils	187 (96.9)
**Surgery**	
No	177 (91.7)
Decompressive craniectomy	5 (2.6)
Craniotomy with clot removal	11 (5.7)
**GOS**	
Death	0
Vegetative state	1 (0.5)
Severe disability	3 (1.6)
Moderate disability	3 (1.6)
Good recovery	186 (96.4)
**Mean VAS scores**	94.08 (9.71)
**Median VAS scores**	98 (90,100)
**Mean EQ-5D-5L index scores**	0.91558 (1.09639)
**Median EQ-5D-5L index scores**	0.93200 (0.068)

Abbreviations: EQ-5D-5L, European quality of life measure-5 domain-5 level; GCS, Glasgow coma scale; GOS, Glasgow outcome scale; IQR, interquartile range; SD, standard deviation; TBI, traumatic brain injury; VAS, visual analogue scale.

**Fig. 1:**
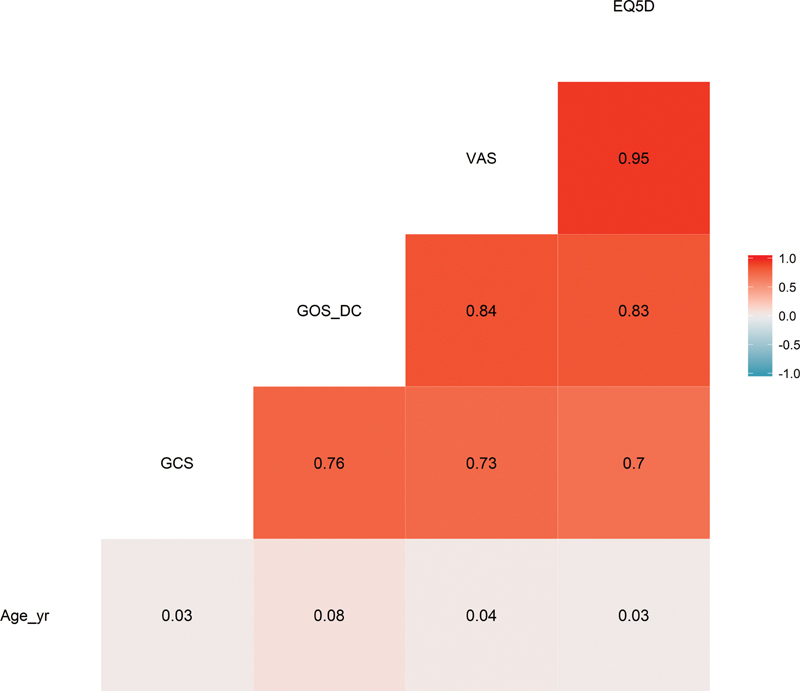
Correlation of the clinical characteristics and European quality of life measure-5 domain-5 level (EQ-5D-5L) index scores.

**Fig. 2:**
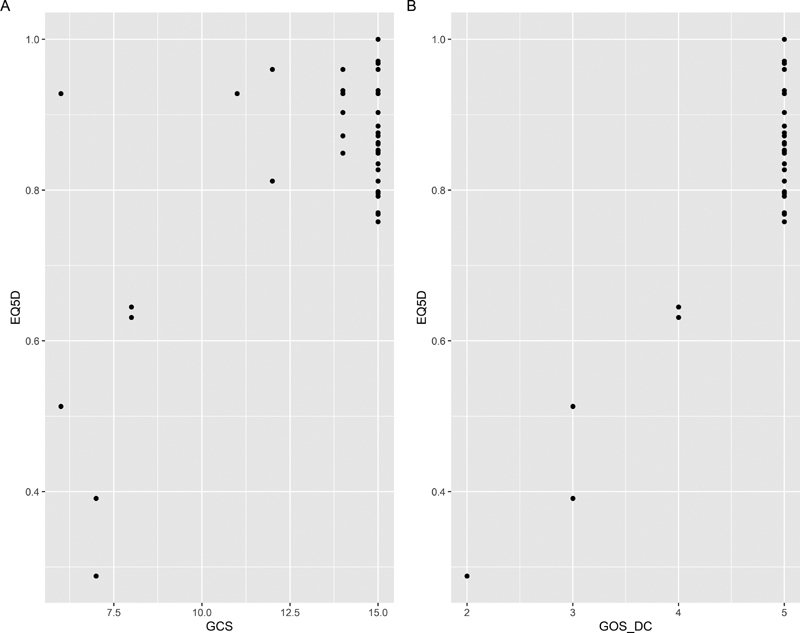
Scatter plot of the European quality of life measure-5 domain-5 level (EQ-5D-5L) index scores with the significant variables. ( **a** ) Glasgow coma scale (GCS) score. ( **b** ) Glasgow outcome scale (GOS) score.

**Fig. 3:**
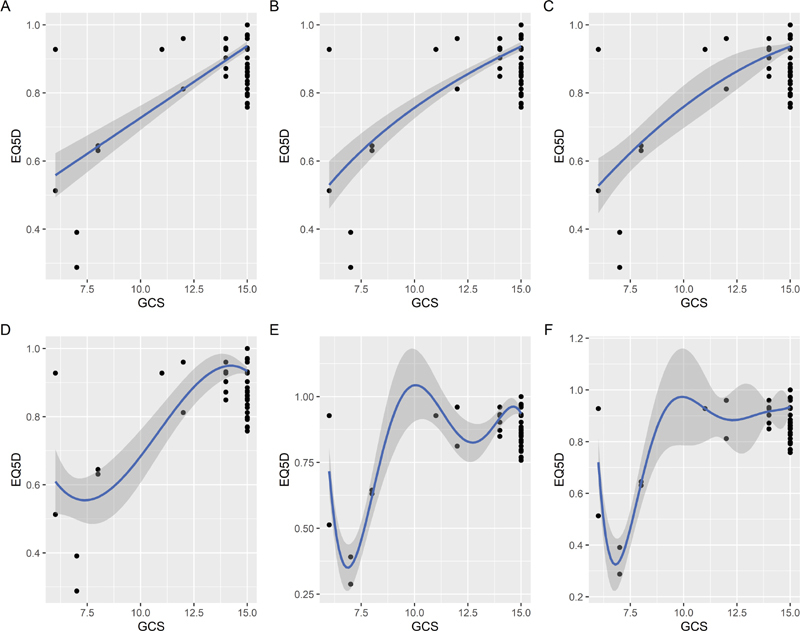
Linear and nonlinear model fitting of the European quality of life measure-5 domain-5 level (EQ-5D-5L) index scores with the Glasgow coma scale score (GCS). ( **a** ) Linear regression, ( **b** ) log transformation, ( **c** ) cubic spline regression, ( **d** ) 3-order polynomial regression, ( **e** ) 5-order polynomial regression, and ( **f** ) 6-order polynomial regression.

**Fig. 4:**
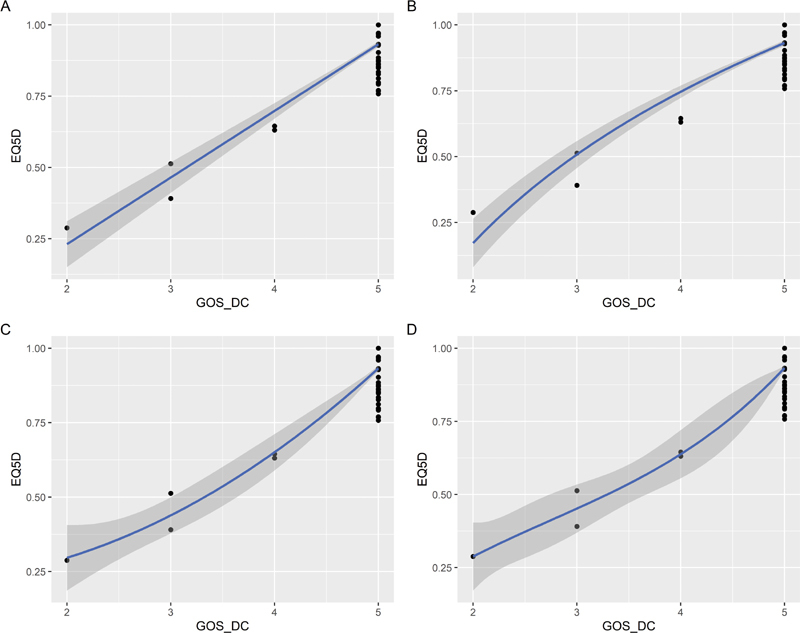
Linear and nonlinear model fitting of the European quality of life measure-5 domain-5 level (EQ-5D-5L) index scores with the Glasgow outcome scale (GOS) score. ( **a** ) Linear regression, ( **b** ) log transformation, ( **c** ) polynomial regression, and ( **d** ) cubic spline regression.

Table 2 shows the mean and median of the EQ-5D-5L index value according to each variable after dividing the data. As a result, the GCS, surgery, pupillary light reflex, and GOS were significantly different from the mean EQ-5D-5L index scores. Additionally, the EQ-5D-5L index score was positively correlated to the GCS score (r = 0.70; *p* < 0.001) and GOS score (r = 0.83; *p* < 0.001) ( Fig. 1 ). The relationship of the EQ-5D-5L index scores and GCS and GOS are shown in ( Figs. 2 a-b ), respectively. The linear and nonlinear regression models were used to establish the relationship between the EQ-5D-5L index scores and other variables ( Figs. 3 a-f for the GCS and Figs. 4 a-d ).

**Table 2:** Mean and median of EQ-5D-5L index scores according to clinical characteristics

Factor	Mean (SD)	Median (IQR)	*p* value a
Age group-year			
< 40	0.91401 (0.13087)	0.93200 (0.075)	0.16
≥ 40	0.91734 (0.08010)	0.92800 (0.806)	
**Gender**			
Male	0.91658 (0.09837)	0.93200 (0.068)	0.60
Female	0.91395 (0.12671)	0.93200 (0.077)	
**Mechanism of injury**			
Road traffic injury	0.93236 (0.057195)	0.93200 (0.068)	0.29
Nonroad traffic injury	0.88013 (0.170241)	0.93000 (0.095)	
**Loss of consciousness**			
No	0.92638 (0.08137)	0.93200 (0.068)	0.26
Yes	0.87554 (0.174980)	0.92800 (0.112)	
**Amnesia**			
No	0.92477 (0.084231)	0.95330 (0.068)	0.58
Yes	0.86359 (0.194499)	0.93200 (0.184)	
**Vomiting**			
No	0.91410 (0.118629)	0.03200 (0.068)	0.56
Yes	0.92123 (0.065496)	0.92800 (0.082)	
**Scalp hematoma/laceration**			
No	0.93429 (0.063533)	0.93200 (0.068)	0.17
Yes	0.90146 (0.132928)	0.93200 (0.088)	
**Bleeding per ear/nose**			
No	0.91441 (0.111364)	0.93200 (0.068)	0.70
Yes	0.94671 (0.033099)	0.93200 (0.040)	
**Hypotension episode**			
No	0.91865 (0.096839)	0.93200 (0.068)	0.75
Yes	0.72100 (0.458357)	0.97100 (0.808)	
**Seizure**			
No	0.91669 (0.109280)	0.93200 (0.068)	0.31
Yes	0.84533 (0.133960)	0.77000 (-)	
**GCS**			
13–15	0.93454 (0.057956)	0.93200 (0.068)	<0.001 b
9–12	0.83371 (0.123323)	0.88500 (0.162)	
3–8	0.51257 (0.249039)	0.51300 (0.357)	
**Pupillary light reflex**			
No react pupils	0.61500 (0.273565)	0.70550 (0.467)	<0.001
React pupils	0.92522 (0.085958)	0.93200 (0.068)	
**Surgery**			
No	0.93153 (0.081419)	0.93200 (0.068)	0.008
Yes	0.73913 (0.199673)	0.79000 (0.306)	
**GOS**			
Vegetative state	0.288000 (-)	0.28800 (-)	<0.001 b
Severe disability	0.36533 (0.162032)	0.39100 (-)	
Moderate disability	0.62533 (0.023029)	0.63100 (-)	
Good recovery	0.93251 (0.059101)	0.93200 (0.068)	

Abbreviations: EQ-5D-5L, European quality of life measure-5 domain-5 level; GCS, Glasgow coma scale; GOS, Glasgow outcome scale.

a *p* value of Mann–Whitney U test.

b *p* value of one-way analysis of variance (ANOVA) test.

Simple linear regression analyses were performed with the clinical variables; therefore, the GCS, surgery, pupillary light reflex and GOS were significant with candidate variables. The four candidate variables were included in the multiple regression model with the backward stepwise procedure. The final model comprised the GOS, surgery and pupillary light reflex variables for the multivariable analysis ( Table 3 ). Also, the multicollinearity of the variables was estimated among the candidate variables. The VIF of the GOS, surgery, and pupillary light reflex was 1.30, 1.39, and 1.44, respectively, while the tolerance of the GOS, surgery, and pupillary light reflex was 0.766, 0.719, and 0.694, respectively. For the predictive performance, the final model had a high adjusted R ^2^ of 0.6971 and a low RMSE of 0.06701. Alternatively, the polynomial regression model of the nonlinear approach had the highest adjusted R ^2^ of 0.6843 and the lowest RMSE of 0.06748. Moreover, the results of the HRQoL obtained by the VAS method were in concordance with the EQ-5D-5L questionnaire method ( Supplementary Material ; available online only).

**Table 3:** Linear and nonlinear fitting between EQ-5D-5L index value and various variables

Factor	Coefficient	*p* value	Adjusted R-squared	RMSE (prediction)
Simple linear regression				
GCS – intercept 0.262	0.045	< 0.001	0.4770	0.08641
Surgery – intercept 0.932	− 0.192	< 0.001	0.2928	0.11330
Pupillary light reflex – intercept 0.615	0.310	< 0.001	0.1364	0.09326
GOS – intercept − 0.370	0.260	< 0.001	0.6790	0.07008
**Multiple linear regression**				
** Full model a**			0.6953	0.06741
Intercept	− 0.302			
GOS	0.230	< 0.001		
Pupillary light reflex	0.102	0.001		
Surgery	− 0.039	0.060		
GCS	0.009	0.852		
** Final model b**			**0.6971**	**0.06701**
Intercept	− 0.302			
GOS		< 0.001		
Pupillary light reflex	0.100	0.001		
Surgery	− 0.038	0.041		
**Nonlinear regression by GOS**				
**Log transformation**			0.6574	0.07462
Intercept	− 0.040131			
Log (GOS)	0.02788	< 0.001		
**Spline regression**			0.6824	0.06819
Intercept	0.28800			
1st knot	0.18192	0.304		
2nd knot	0.26671	0.068		
3rd knot	0.64475	< 0.001		
**Polynomial**			**0.6843**	**0.06748**
Intercept	0.9166			
1st order	1.00265	< 0.001		
2nd order	0.10085	< 0.001		

Abbreviation: GCS, Glasgow coma scale; GOS, Glasgow outcome scale; IQR, interquartile range; RMSE, root mean square error; SD, standard deviation; VIF, variance inflation factors.

a VIF of GOS, pupillary light reflex, surgery, and GCS were 2.38, 1.55, 1.85, and 3.84, while tolerances of those were 0.419, 0.643, 0.538, and 0.260, respectively.

b VIF of GOS, pupillary light reflex, and surgery were 1.30, 1.39, and 1.44, while tolerances of those were 0.766, 0.719, and 0.694, respectively.

## Discussion

Poor functional outcomes among TBI patients have been observed in 0.6 to 24.3%, while mortality ranged from 19.5 to 29.4%. 35 36 Not only did mortality and morbidity have a direct impact on the individuals suffering from TBI, but long-term neurological sequelae were also socioeconomic burdens that should be of concern. 10 11 12 HRQoL is one of the health outcomes which was measured according to the patients' perspective according to the concept of holistic medicine. 37

Although the EQ-5D-5L questionnaire is widely used to measure HRQoL, this tool is specifically built based on European measures. The socioeconomic and cultural issues vary in the eastern population which may affect the use of the tool. Pattanaphesaj et al conducted a survey of HRQoL in the Thai general population and developed a Thai value set for EQ-5D-5L which became the guidelines in Thailand. 28 32 Therefore, we used this value set for HRQoL measurement in the present study. In the results, the GOS was a strong predictor associated with the HRQoL in which the concordance results were similar to those of prior reports. Kosty et al studied the relationship between the GOS-E with the HRQoL by using the standard gamble approach in 101 patients and demonstrated that there was a strong correlation between the GOS-E with the HRQoL (R ^2^ = 0.637; *p* < 0.001). 38 Also, the mean HRQoL of severe disability based on the GOS was less than the moderate disability and good recovery categories from Tsauo et al. 39

From the literature review, a few studies mentioned HRQoL and the associated factors in TBI. Therefore, Ward et al developed a predictive model from the EQ-5D-5L index scores weighted on the GOS, age at injury, sex, comorbidity, and major extracranial injury from a systematic review and mapping approach. A linear relationship between the mean EQ-5D-5L values and GOS were described in the study. 14 26 From the present study, the author developed a favorable model predicting the EQ-5D-5L index scores based on the GOS, surgery, and pupillary light reflex.

The GCS was one of the predictors in the univariate analysis. Although the GCS is significantly correlated with the GOS (r = 0.076; *p* < 0.001), the VIF and tolerance of the GCS variables in the multivariable analysis were not out of range, so there was no multicollinearity in the full model. However, the GCS had a low performance for prediction; hence, the GCS variable was finally removed during the selection of the model. This result may have occurred from an imbalance in the numbers of the GCS which were mainly 13 to 15 in the present cohort. Therefore, compared with previous studies, the present finding was in concordance with the literature. 40 41 Kodliwadmath et al studied 82 TBI patients and found that there was a statistically significant positive correlation between the GCS on admission with the GOS documented on day 7 (r = 6.19) and the GOS listed on day 28 (r = 5.77). 41

Although linear regression has been used for predicting the HRQoL, 40 42 43 44 the present study proposed alternative approaches for predicting the EQ-5D-5L index scores by a nonlinear model. To the author's knowledge, this was the first study that had revealed polynomial regression with acceptable performance to predict the EQ-5D-5L index scores using a single variable like the GOS.

The limitations of the present study were also acknowledged. First, an imbalance in the number of patients with moderate-to-severe TBI was detected in the current cohort. Moderate-to-severe TBI patients would need a longer time for recovery than the remaining group. Assessment of the outcome and HRQoL in the long-term follow-up may also demonstrate more informative results. Moreover, a meta-analysis or multicenter study should be conducted in the future to increase the number of the study population. Likewise, the present study's predictive models would need to be tested and improve their performance by external validation with unobserved data. 45 Second, the observational study design resulted in a bias from the confounders. Although the multivariable analysis could control the confounding factors, nowadays the propensity score approach is an alternative way to resolve this problem for a nonrandomized controlled trial. 46 Third, prior studies were concerned that the GOS might lead to a ceiling effect and the GOS-E was replaced for the assessment of the outcome. However, the sample size should be further enlarged for testing the hypothesis when using the eight-scale GOS-E.

Finally, the assessment of the GOS and HRQoL were estimated only before the participants were discharged from hospital. Prior studies had principally estimated the patients' outcome within 6 months. 40 On the other hand, posttraumatic complications such as postconcussion syndrome were found within the first week to 3 months 47 48 and affected the HRQoL. 49 Hence, long-standing assessments of the outcome and the HRQoL should be conducted in the future for long-term results.

## Conclusions

In summary, the current study revealed a positive relationship between physician-based outcome (GOS) and patient-based outcome (HRQoL). Moreover, both the linear and nonlinear regression models were acceptable approaches to predict the HRQoL of patients after TBI. There would be limitations to obtain the HRQoL from unconsciousness or intubated patients directly; therefore, this would be a challenge to use the predicted values for estimating HRQoL.
