# Supplementary material for: Predicting the Health-related Quality of Life in Patients Following Traumatic Brain Injury
Source: Surg J (N Y). 2021 Jun 17;7(2):e100–10. doi: 10.1055/s-0041-1726426 (PMC8211484; doi:10.1055/s-0041-1726426)
Supplement: Supplementary file 1 — Supplementary Material [file 10-1055-s-0041-1726426-s2000105oa.pdf]

Supplementary Material

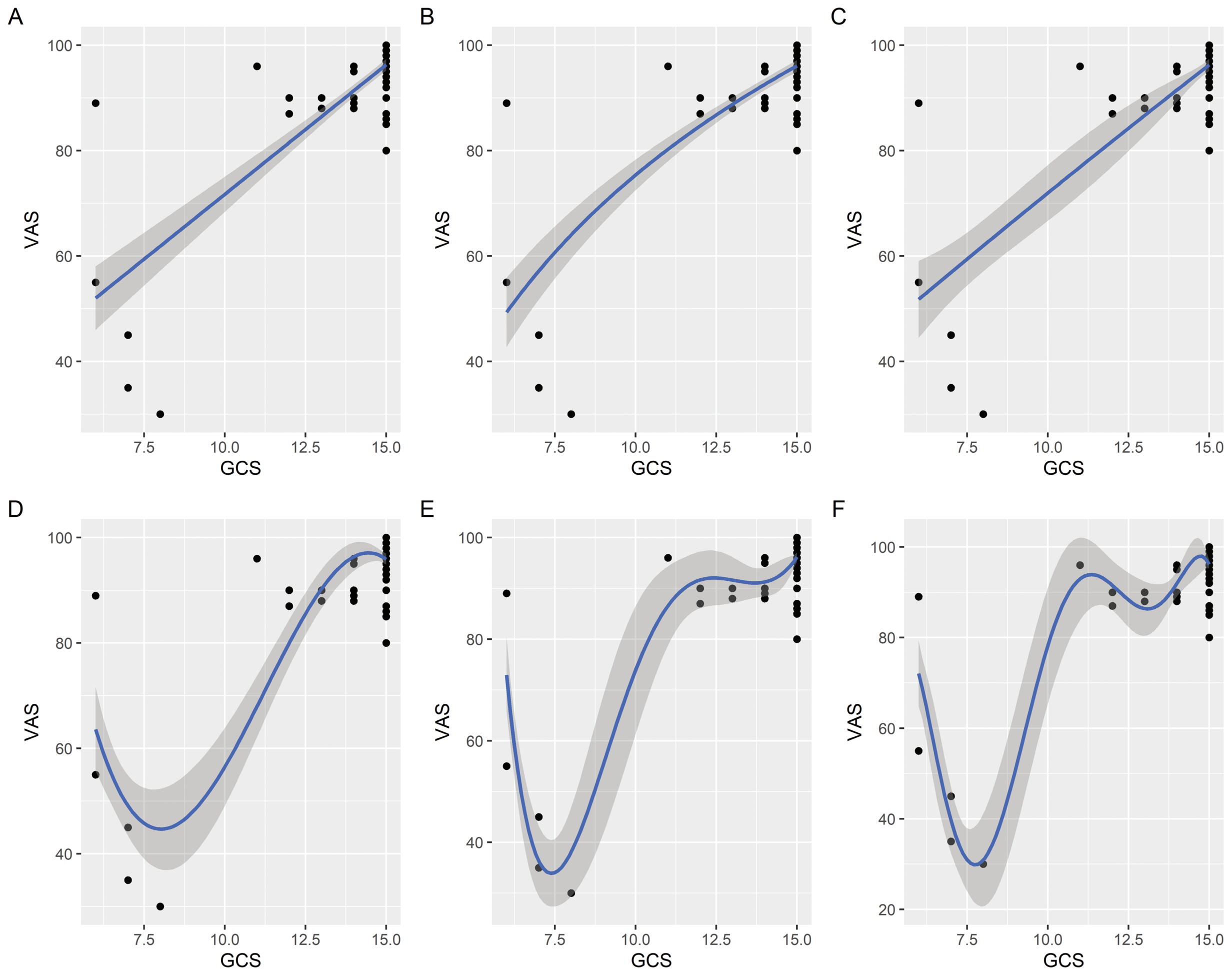

Supplementary Figure 1 Linear and non-linear model fitting of the health-related quality of life by visual analogue scale (VAS) by Glasgow Coma Scale score (GCS). (A) Linear regression. (B) log transformation. (C) cubic spline regression. (E) 3-order polynomial regression. (E) 5-order polynomial regression. (F) 6-order polynomial regression.

 © 2021. The Author(s).

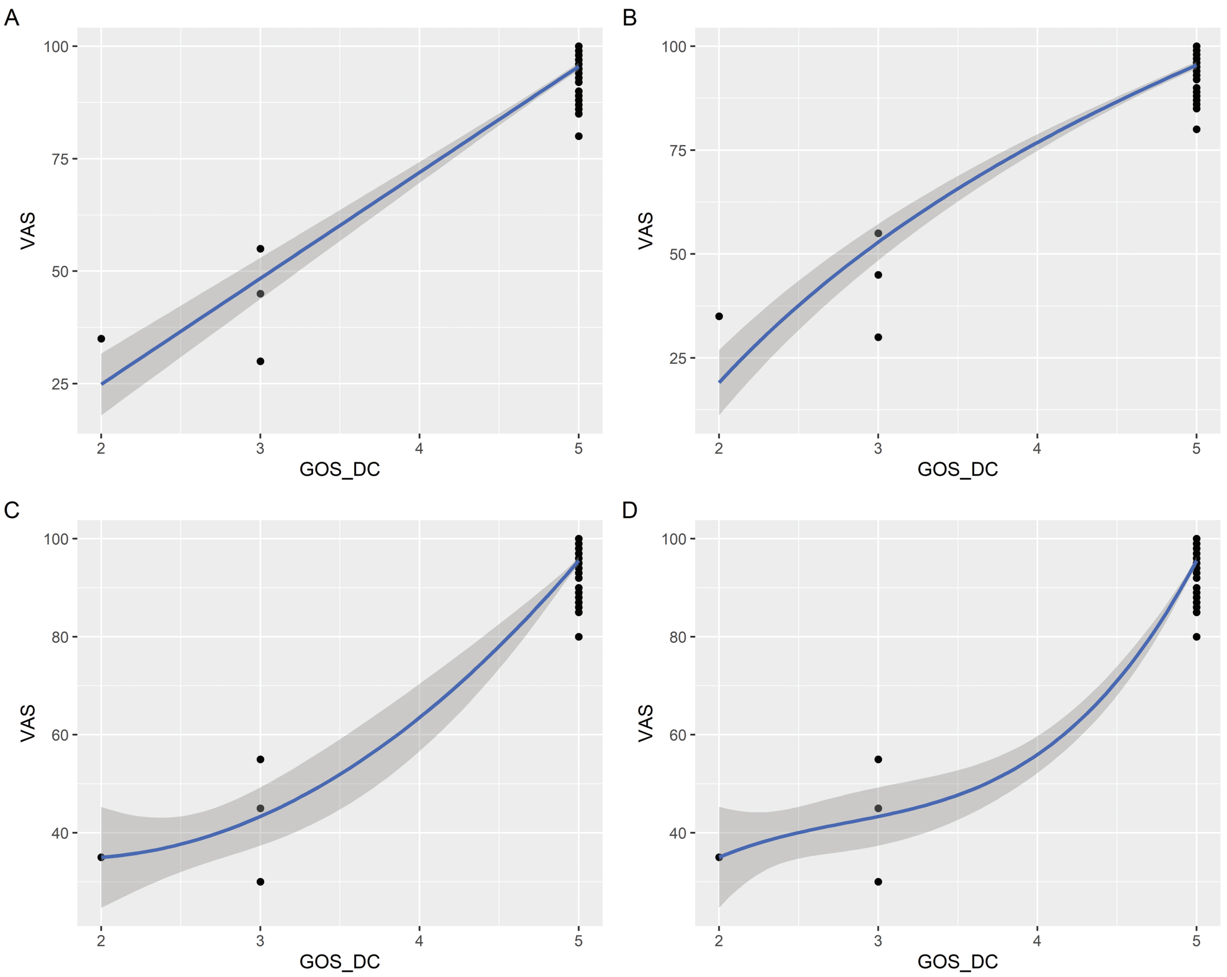

Supplementary Figure 2 Linear and non-linear model fitting of the health-related quality of life by visual analogue scale (VAS) by Glasgow Outcome Scale score (GOS_DC). (A) Linear regression. (B) log transformation. (C) polynomial regression. (D) cubic spline regression.

 © 2021. The Author(s).

Supplementary Table 1 Mean and median of the HRQoL by VAS method according to clinical characteristics

Factor	Mean (SD)	Median (IQR)	*p* value[a]
**Age group-year**			0.51
< 40	93.77 (11.59)	98 (10)	
≥ 40	94.43 (7.07)	97 (10)	
**Gender**			0.34
Male	94.10 (8.47)	98 (10)	
Female	94.05 (11.17)	98 (10)	
**Mechanism of injury**			0.14
Road traffic injury	95.67 (5.00)	98 (10)	
Nonroad traffic injury	90.74 (15.05)	98 (10)	
**Loss of consciousness**			0.29
No	95.06 (7.07)	98 (10)	
Yes	90.46 (15.69)	97 (10.5)	
**Amnesia**			0.47
No	94.93 (7.37)	98 (10)	
Yes	89 (17.45)	97 (13)	
**Vomiting**			0.61
No	93.92 (10.54)	98 (10)	
Yes	94.72 (5.49)	96.5 (10)	
**Scalp hematoma/laceration**			0.24
No	95.74 (5.16)	98 (10)	
Yes	92.83 (11.93)	96 (10)	
**Bleeding per ear/nose**			0.67
No	94.00 (9.84)	98 (10)	
Yes	96.28 (4.78)	98 (10)	
**Hypotension episode**			0.78
No	94.36 (8.57)	98 (10)	
Yes	76.66 (40.41)	100 (70)	
**Seizure**			0.24
No	94.17 (9.70)	98 (10)	
Yes	88.33 (10.40)	85 (20)	
**GCS**			<0.001†
13–15	95.81 (4.90)	98 (10)	
9–12	86.57 (9.05)	88 (15)	
3–8	57.42 (22.3)	55 (43)	
**Pupillary light reflex**			<0.001
No react pupils	67.00 (23.64)	79 (23.64)	
React pupils	94.95 (7.60)	98 (10)	
**Surgery**			<0.001
No	95.54 (7.02)	98 (10)	
Yes	77.93 (18.01)	83.5 (17.75)	
**GOS**			< 0.001[b]
Vegetative state	35	35	
Severe disability	43.33 (12.58)	45 (-)	
Moderate disability	72.66 (4.61)	70 (-)	
Good recovery	95.56 (5.06)	98 (10)	

Abbreviations: GCS, Glasgow coma scale; GOS, Glasgow outcome scale; HRQoL, health-related quality of life; IQR, interquartile range; SD, standard deviation; VAS, visual analogue scale.
[a]*p* value of Mann–Whitney U test.
[b]*p* value of one-way analysis of variance (ANOVA) test.

 © 2021. The Author(s).

Supplementary Table 2 Linear and nonlinear fitting between the HRQoL by VAS and various variables

Factor	Coefficient	*p* value	Adjusted R-squared	RESM (prediction)
**Simple linear regression**				
GCS (intercept + 33.26)	+ 4.20	< 0.001	0.6172	5.8978
Surgery (intercept + 95.54)	− 17.61	< 0.001	0.4055	6.1995
Pupillary light reflex (intercept + 67.00)	+ 27.95	< 0.001	0.1915	7.7242
GOS (intercept − 21.86)	+ 23.48	< 0.001	0.6001	5.0779
**Multiple linear regression**				
**Full model**[a]			0.6262	5.1211
Intercept	− 17.50			
GOS	+ 19.99	< 0.001		
Pupillary light reflex	+ 11.75	< 0.001		
Surgery	− 3.30	0.08		
GCS	+ 0.112	0.82		
**Final model**[b]			**0.6318**	**5.0992**
Intercept	− 17.45			
GOS	+ 20.26	< 0.001		
Pupillary light reflex	+ 12.01	< 0.001		
Surgery	− 3.48	0.041		
**Nonlinear regression by GOS**				
**Log transformation**			**0.6001**	**5.2130**
Intercept	− 38.72			
Log (GOS)	+ 83.37	< 0.001		
**Spline regression**			0.6001	6.9092
Intercept	+ 35.00			
1st knot	+ 13.70	0.29		
2nd knot	NA	NA		
3rd knot	+ 60.52	< 0.001		
**Polynomial**			0.6001	5.6900
Intercept	94.13			
1st order	106.56	< 0.001		
2nd order	13.42	< 0.001		

Abbreviation: GCS, Glasgow coma scale; GOS, Glasgow outcome scale; HRQoL, health-related quality of life; IQR, interquartile range; NA, not applicable; RMSE, root mean square error; SD, standard deviation; VAS, visual analogue scale; VIF, variance inflation factors.
[a]VIF of GOS, pupillary light reflex, surgery, and GCS were 2.38, 1.55, 1.85, and 3.84, while tolerances of those were 0.419, 0.643, 0.538, and 0.260, respectively.
[b]VIF of GOS, pupillary light reflex, and surgery were 1.30, 1.39, and 1.44, while tolerances of those were 0.766, 0.719, and 0.694, respectively.

 © 2021. The Author(s).
